# Therapeutic Failure or Success Risk Associated With the Dynamic of Immunological Parameters in People Living With HIV‐1 on Antiretroviral Treatment in Benin

**DOI:** 10.1155/arat/1119922

**Published:** 2026-08-30

**Authors:** Yaou Pierrot Assogba, Odilon Paterne Nouatin, Edmond Tchiakpe, Adefounke Prudencia Adechina, Rufine Abossèdé Fachinan, Roukoyath Moyoriola Adegnika, Euloge Sènan Adjou, Halimatou Diop-Ndiaye, Honoré Sourou Bankole, Akadiri Yessoufou

**Affiliations:** ^1^ Laboratory of Integrative Biology for Therapeutic Innovation (BioInov), Unit of Cellular Biology and Immunology of Chronic and Infectious Diseases (UBCI-MCI), Faculty of Sciences and Technology (FAST), University of Abomey-Calavi (UAC), 01 BP 526, Cotonou, Benin, uac.bj; ^2^ Clinical Research Institute of Benin, Abomey-Calavi 04 BP 1114, Cotonou, Benin; ^3^ National Reference Laboratory of Health Program Fighting Against AIDS in Benin (LNR/PSLS), Ministry of Health, BP 1258, Cotonou, Benin, sante.gov.ma; ^4^ Lambaréné Medical Research Center (CERMEL), BP 242, Lambaréné, Gabon; ^5^ Laboratory of Bacteriology-Virology of CHU Aristide Le Dantec Hospital, University Cheikh Anta Diop of Dakar, BP 5005, Dakar, Senegal; ^6^ Research Unit in Applied Microbiology and Pharmacology of Natural Substances (URMAPHA), EPAC, University of Abomey-Calavi (UAC), 01 BP 2009, Cotonou, Benin, uac.bj; ^7^ Tropical Infectious Diseases Research Center (TIDRC), University of Abomey-Calavi (UAC), 01 BP 526, Cotonou, Benin, uac.bj; ^8^ Institute of Applied Biomedical Sciences (ISBA), Ministry of High Education and Scientific Research, 01 BP 918, Cotonou, Benin

**Keywords:** Benin, cytokines, dolutegravir, efavirenz, immunes cells, PLHIV-1, therapeutic risk factors

## Abstract

Until now, human immunodeficiency virus type 1 infection monitoring is based on plasma viral load and cluster of differentiation (CD)4–positive cells from thymus (CD4+ T cells) count. However, it is increasingly accepted that this biological monitoring should be strengthened with other markers that would reinforce the management. Therefore, we addressed this task by assessing the risk associated with therapeutic failure/success linked to the dynamic of immunological parameters in people living with HIV‐1 (PLHIV‐1) and on antiretroviral therapy (ART) in order to identify their prognostic values. Ninety enrolled PLHIV‐1 were classified according to their therapeutic status. Twenty healthy persons were also recruited as a control group. Serum cytokine levels and immune cell frequencies were determined, and the risk of therapeutic failure associated with immunological parameters was assessed. We observed low frequencies of CD4+ T, natural killer (NK), natural killer T (NKT) cells, classical monocytes, nonclassical monocytes, granulocytes, and high frequency of CD8+ T cells in all PLHIV‐1 groups in treatment failure under dolutegravir (DTG) and efavirenz (EFV) regimens compared to control participants. Moreover, interferon‐gamma (IFN‐γ) levels decreased in PLHIV‐1 with therapeutic success under the DTG regimen, while interleukin‐4 (IL‐4) increased in treatment success under the EFV regimen. Proinflammatory tumor necrosis factor‐alpha (TNF‐α), IL‐6, and IL‐7 significantly increased in therapeutic failure groups under both regimens. IL‐5 concentrations increased in all PLHIV‐1, while IL‐13 levels did not change. Logistic regression analysis revealed a positive correlation between the risk of treatment failure and proinflammatory cytokines IFN‐γ, TNF‐α, IL‐6, IL‐7, eosinophils, and CD8+ T cells in PLHIV‐1 under EFV and DTG regimes. In contrast, the risk of therapeutic failure decreased with increasing numbers of CD4+ T cells, neutrophils, and the anti‐inflammatory IL‐4 in PLHIV‐1 treated with the same antiretroviral molecules. Collectively, these results indicated that pro‐ and anti‐inflammatory cytokines and immune cells could serve as prognostic factors for monitoring HIV‐1 disease progression and response to ART. However, further studies with a larger sample size would be needed to confirm our data.

## 1. Introduction

Human immunodeficiency virus Type 1 (HIV‐1) infection is characterized by progressive loss of CD4+ T lymphocytes concomitant with the disruption of the immune status of PLHIV‐1 linked to immune cells’ functions, such as CD8+ cytotoxic T cells, dendritic cells, neutrophils, monocytes/macrophages, and natural killer (NK) cells [1, 2]. In general, in HIV‐1 infection, serum cytokine levels increase with immunosuppression, whereas they decrease after antiretroviral therapy (ART), which contributes to the restoration of immune cell function and number [3, 4]. Thus, by inhibiting HIV‐1 replication, ART reverses the immunopathological mechanisms resulting from HIV‐1 infection [5]. This phenomenon severely impacts the immune status of PLHIV‐1, as these CD4+ T lymphocytes, through their cytokines, exert distinct biological functions in controlling immune system homeostasis [6]. Several studies have shown that immune function can be altered by both down‐ or upregulation of T helper 1 (Th1) and T helper 2 (Th2) cytokines, respectively, during HIV‐1 infection [7, 8]. Very recently, we have demonstrated that NK and NKT cells, along with IL‐6, TNF‐α, IL‐5, and IL‐7 cytokines, could serve as valuable immunological biomarkers in the therapeutic monitoring of PLHIV‐1, in addition to the traditional CD4/CD8 T‐cell ratio [9]. It has been demonstrated that ART can regulate the Th cell cytokine production in HIV infection [8, 10].

Therapeutic approaches consisted of the combined use of two nucleoside reverse transcriptase inhibitors (NRTI) and a non‐nucleoside reverse transcriptase inhibitor (NNRTI) or an integrase inhibitor (IIN) or a protease inhibitor (PI) [11]. The role of these molecules is to inhibit viral replication with profound beneficial changes in viral and cellular dynamics and immune status [5]. However, the different classes of antiretrovirals (ARVs) have shown different effects on immune cell functions and inflammatory biomarkers [12]. Indeed, PIs such as ritonavir prevented HIV‐1 replication induced by TNF‐α, IL‐1β, and IL‐6 [13], whereas a therapeutic regimen including reverse transcriptase inhibitors down‐modulated the production of cytokines such as IL‐6, TNF‐α, IFN‐γ, and IL‐12 produced by HIV‐1‐infected monocytes [14, 15]. IINs induced a decrease in biomarkers of inflammation and immune activation by restoring CD8+ T lymphocyte levels compared to other classes of ARVs [16, 17].

In Benin, the first‐line treatment consists of two NRTIs and one NNRTI or an IIN, tenofovir plus lamivudine plus efavirenz/dolutegravir (TDF + 3TC + EFV/DTG). Thus, most PLHIV‐1 adults are placed on this first‐line alternative, which combines TDF + 3TC + EFV or TDF + 3TC + DTG [18]. These molecules acted to interfere in various ways with the mechanisms of viral replication and the activation of immune cells responsible for the depletion of CD4+ T cells and CD8+ T cells [19]. Recent studies in Africa reported that the use of TDF + 3TC + EFV could inhibit the viral replication process and promote an increase in the number of CD4 T lymphocytes [20, 21]. Conversely, other authors showed that this previously cited therapeutic regimen induced virologic and immunological failure of 11.34% and 37.17%, respectively, in PLHIV‐1 [22], while a regimen based on TDF/3TC/DTG presented a more favorable profile in terms of viral suppression and immunological recovery than a regimen based on tenofovir/lamivudine/EFV [23].

For several decades, the effectiveness of these molecules in PLHIV‐1 has been measured by clinical criteria or standardized surrogate markers, notably CD4+ T lymphocyte number and plasma viral load (VL) [24, 25]. However, CD4+ T cells–based immunological and clinical monitoring has lower positive predictive value and reduced sensitivity for identifying treatment failure compared to viral suppression [21]. It then appeared necessary to explore other immunological factors associated with therapeutic failure or success in PLHIV‐1, depending on different classes of ARV molecules, in order to identify relevant alternative monitoring biomarkers with prognostic value. In this perspective, the present study aims to identify new immunological markers possessing prognostic values to monitor the progression of HIV infection. To do this, we evaluated the risk factors associated with therapeutic failure or success linked to the dynamics of immune cells and cytokines in a first‐line therapeutic regimen combining TDF + 3TC + EFV or TDF + 3TC + DTG in Benin.

## 2. Subjects

### 2.1. Study Area and Design

A cross‐sectional study was conducted at the Abomey‐Calavi/Sô‐Ava University Hospital (Benin). This public secondary‐level healthcare facility serves a large population in the Abomey‐Calavi and Sô‐Ava municipalities and plays a key role in the decentralized management of HIV infection. The hospital provides comprehensive HIV care, including voluntary testing, ART initiation, and routine clinical follow‐up. The study included patients attending routine monitoring visits during the study period.

### 2.2. Study Population

The study population consisted of PLHIV‐1 receiving ART for at least 6 months and monitored at the study site by the specialist clinicians. Participants were considered eligible and included in the study if they were aged at least 18 years old, not pregnant, and not receiving anti‐inflammatory treatment, had a confirmed infection with HIV‐1 only, and consented to attend routine visits at the study site during the study period.

Participants who were pregnant or coinfected with HIV‐1/HIV‐2, or had a history of diabetes mellitus, or showing abnormal blood cell counts, or presenting opportunistic infections, such as diarrhea, malaria, or parasitic diseases, were excluded from the study. Indeed, participants were excluded if they were pregnant, as pregnancy is associated with physiological and immunological changes that could alter the study parameters. Individuals receiving anti‐inflammatory treatment were also excluded because such treatments may interfere with inflammatory and biological markers. Furthermore, an abnormal blood cell count in patients usually suggests infection or other underlying conditions. Such patients were excluded in order to avoid potential confounding factors. Individuals with a history of diabetes mellitus were excluded due to the known impact on metabolic and immune parameters. Finally, participants presenting opportunistic infections such as diarrhea, malaria, or parasitic diseases were excluded to minimize bias related to altered immune status and viral dynamics.

Participants were consecutively recruited among eligible PLHIV‐1 during their routine monitoring visits at the hospital during the study period. They were divided into four groups: PLHIV‐1‐DTGs (*n* = 25), patients receiving TDF + 3TC + DTG with therapeutic success and an undetectable VL < 40 copies/mL; PLHIV‐1‐DTGf (*n* = 13), patients on the same regimen with therapeutic failure and VL > 1000 copies/mL; PLHIV‐1‐EFVs (*n* = 15), patients receiving TDF + 3TC + EFV with therapeutic success; and PLHIV‐1‐EFVf (*n* = 37), patients on TDF + 3TC + EFV with therapeutic failure. Twenty healthy participants seronegative for HIV infection were recruited and considered as the control group. No random sampling was performed. No formal matching between groups was conducted. The unequal distribution of participants across groups reflects the real‐life clinical outcomes and treatment responses observed in the study population.

Sociodemographic data, along with some clinical and biological data of the participants, were recorded and summarized in Table 1. The therapeutic success is defined as achieving an undetectable HIV‐1 VL (below 40 copies/mL) in all patients receiving ART after six months of treatment, while therapeutic failure is defined as detectable HIV‐1 VL after two consecutive determinations showing VL greater than 1000 copies/mL at three‐month intervals in all patients receiving ART during the same period.

**TABLE 1:** Clinical data and sociodemographic characteristics of control participants and PLHIV‐1 who were on ARV for at least 6 months in the study cohort.

Characteristics	Healthy control participants	PLHIV‐1‐DTGs	PLHIV‐1‐DTGf	PLHIV‐1‐EFVs	PLHIV‐1‐EFVf	*p* value
Number of participants	20	25	13	15	37	
Sex category						
Female *n* (%)	9 (45)	18 (72)	9 (69)	10 (67)	28 (77)	—
ARV regimen	—	TDF + 3TC + DTG	TDF + 3TC + DTG	TDF + 3TC + EFV	TDF + 3TC + EFV	
Median age [IQR]	39.5 [35.25–43]	45 [37.5–49.50]	40 [32.5–52]	45 [33–58]	40 [37–49.5]	^b^ *p* = 0.77
Median viral load (log_10_) [IQR]	Not applied	Undetectable	4.33 [3.32–5.79]	Undetectable	4.65 [3.98–5.12]	^a^ *p* = 0.59
Median ARV treatment duration (months) [IQR]	—	24.28 [4.31–29.83]	22.67 [15.87–63.69]	75.45 [37.97–84.53]	53.08 [37.33–77.16]	^b^ *p* = 0.005

*Note:* TDF: tenofovir, 3TC: lamivudine, EFV: efavirenz, DTG: dolutegravir, IQR: Interquartile. Healthy control participants; *n* = 20; PLHIV‐1‐DTGs: People living with HIV‐1 on DTG regimen (TDF + 3TC + DTG) and with therapeutic success; *n* = 25; PLHIV‐1‐DTGf: People living with HIV‐1 on DTG regimen (TDF + 3TC + DTG) and with therapeutic failure; *n* = 13; PLHIV‐1‐EFVs: People living with HIV‐1 on EFV regimen (TDF + 3TC + EFV) and with therapeutic success; *n* = 15; PLHIV‐1‐EFVf: People living with HIV‐1 on EFV regimen (TDF + 3TC + EFV) and with therapeutic failure; *n* = 37. The statistical differences were determined using the nonparametric Kruskal–Wallis test and Mann–Whitney test. Differences were considered significant when *p* values are less than 0.05.

^a^Mann–Whitney test between PLHIV‐1f group under TDF + 3TC + DTG and PLHIV‐1f group under TDF + 3TC + EFV.

^b^Kruskal–Wallis test between healthy controls and the groups of therapeutic success or failure according to the treatment line, or between the therapeutic success or failure according to the treatment line.

## 3. Methods

Immunological assays were performed at the Unit of Cellular Biology and Immunology of Chronic and Infectious Diseases at the BioInov Lab, Department of Biochemistry and Cellular Biology, Faculty of Sciences and Technology (FAST), University of Abomey‐Calavi (UAC). Virologic assays were performed in collaboration with the National Reference Laboratory of the Health Program Fighting Against AIDS in Benin (LNR/PSLS). The flow cytometry device, FACS Canto‐II, has been provided by the laboratory of the Institut de Recherche Clinique du Bénin (IRCB), Calavi, Benin.

### 3.1. Sample Collection and Storage

A quantity of 2.5 mL of venous whole blood was collected in EDTA tubes and transported to the laboratory in refrigerated coolers. Then, 300 μL of whole blood was systematically used for immune cell phenotyping. Plasma isolated after centrifugation at 3500 rpm for 20 min was used for the determination of plasma VL at the Reference Laboratory of the National AIDS Health Program. Serum obtained from blood collected in a dry tube after centrifugation was used for cytokine quantification.

### 3.2. Plasma VL Measurement

Plasma HIV‐1 RNA VL was performed using Cobas TaqMan 96/Cobas Ampliprep (CAP/CAP‐CTM) HIV‐1 equipment (Roche Molecular Diagnostics, Basel, Switzerland), according to the manufacturer’s instructions and as we have previously described [9, 26].

### 3.3. Immune Cell Phenotyping

Leukocyte subpopulations were determined by flow cytometry after cell staining with monoclonal antibodies as in the recent study carried out by Assogba et al. [9]. Monoclonal antibodies purchased from BD Pharmingen (France) were used for cell labeling in whole blood. In summary, a first combination of monoclonal antibodies (3 μL each of anti‐CD3‐PerCP, anti‐CD4‐FITC, anti‐CD8‐PE, and anti‐CD56‐APC) was prepared to identify, respectively, CD4+ and CD8+ T lymphocytes, NK, and NKT cells. A second combination of 3 μL each monoclonal antibody (anti‐CD3‐PerCP, anti‐CD14‐FITC, anti‐CD16‐APC/Cy7, and anti‐CD19‐PE) was used to characterize monocytes, B lymphocytes, and polymorphonuclear neutrophils (PNNs) and eosinophils (PNE). Briefly, a quantity of 300 μL of whole blood was incubated with an optimized volume of antibodies (3 μL each) for 30 min at 4°C in the dark. Then, FACS‐Lysing‐1X buffer (2 mL) was added to each tube to lyse red blood cells. After all, the mixture was incubated in the dark for 10 min at room temperature and washed twice with 1X PBS (3 mL per tube) by centrifuging for 8 min, at 1000 rpm at 4°C. The stained cells were then suspended in 300 μL of 1X PBS. Acquisition of 100,000 cells from each tube was done with the BD FACS Canto‐II flow cytometer. The results were analyzed with Flow‐Jo 7.6 Version 10.8.1 software (BD Pharmingen, France). Gating strategies were used to identify immune cell subpopulations. Total lymphocytes’ population frequency was obtained based on forward scatter (FSC) versus side scatter (SSC) techniques of a specific laser wavelength on the cells. NK cells (CD3− CD56+) and NKT cells (CD3+ CD56+) were obtained by gating from total lymphocytes. The CD4+ and CD8+ T cells were obtained from CD3+ CD56− T cells. The total B cells (CD19+) were gated from total lymphocytes using CD3 and CD19 monoclonal antibodies. The monocyte and granulocyte populations were gated based on FSC‐A and SSC‐A in blood cells. Nonclassical monocytes (CD16++ CD14+), intermediate monocytes (CD14++ CD16+), and classical monocytes (CD14++ CD16−) were gated from total monocytes using CD14 and CD16 monoclonal antibodies, whereas neutrophils and eosinophils were identified based on CD16 in polymorphonuclear cells [9].

### 3.4. Serum Cytokine Measurements by ELISA

Serum cytokine levels were measured by sandwich ELISA as in the recent study carried out by Adechina et al. [26]. IFN‐γ (known as Th1 cytokine), IL‐4 (known as Th2 cytokine), and TNF‐α, IL‐5, IL‐6, and IL‐7 (as proinflammatory cytokines), as well as IL‐13 (as anti‐inflammatory cytokine), were quantified using Human Peprotech ELISA kits (Rocky Hill, NJ 08553 USA), according to the manufacturer’s instructions. Briefly, standards and diluted samples were added to precoated 96‐well plates and incubated to allow antigen binding. After the washing steps, a biotinylated detection antibody and enzyme conjugate were added, followed by a substrate solution for color development. The reaction was stopped, and optical density was measured using a microplate reader at the recommended wavelength. Cytokine concentrations were calculated from standard curves generated with known standards.

### 3.5. Statistical Data Analysis

Statistical analyses were performed using R software (Version 4.1.2). Graphs were made using the GraphPad software (Version 8.3.0). The comparison of cytokine concentrations and cell frequencies between the control and cases (HIV‐1‐positive) group was done using the Mann–Whitney test, and between participants’ groups following each treatment line or compared to the control group was performed using the Kruskal–Wallis test with Dunn’s multiple comparisons test. The logistic regression analysis was performed to assess the risk factors associated with antiretroviral treatment failure. The numeric values were divided into two groups according to the value below the medium (low), and values above the medium (high). The categories “medium (low)” and “medium (high)” were determined based on the median value of the variable, calculated using R statistical software*.* *p* values less than 0,05 were considered as statistically significant.

## 4. Results

### 4.1. Characteristics of the Study Population

PLHIV‐1 were on two lines of treatment, but the preponderant line was TDF + 3TC + EFV (70.83%), as shown in Table 1. The proportion of female participants was as follows: 45% in the healthy control group, 72% in PLHIV‐1‐DTGs, 69% in PLHIV‐1‐DTGf, 67% in PLHIV‐1‐EFVs, and 77% in PLHIV‐1‐EFVf groups were women. The median age of participants in each group was 39.4 [35.25–43] years for the healthy control group, 45 [37.5–49.50] years for PLHIV‐1‐DTGs, 40 [32.5–52] years for PLHIV‐1‐DTGf, 45 [33–58] years for PLHIV‐1‐EFVs, and 40 [37–49.5] years for PLHIV‐1‐EFVf. The median VL was 4.33 [3.32–5.79] Log_10_ in PLHIV‐1 under DTG with treatment failure, and 4.65 [3.98–5.12] Log_10_ in PLHIV‐1 under EFV with treatment failure, while it was undetectable in PLHIV‐1 with treatment success, as expected. The average treatment duration (in month) was 24.28 [4.31–29.83], 22.67 [15.87–63.69], 75.45 [37.97–84.53], and 53.08 [37.33–77.16] in PLHIV‐1‐DTGs, PLHIV‐1‐DTGf, PLHIV‐1‐EFVs, and PLHIV‐1‐EFVf, respectively, showing a difference in ART duration between the PLHIV groups.

### 4.2. Global Frequencies of Immune Cells and Cytokine Concentration in PLHIV‐1 Compared to the Control

Regardless of the treatment line, global analyses showed that the frequencies of total CD4+ T cells (*p* < 0.0001), NK (*p* < 0.0001), NKT (*p* < 0.0001), B cells (*p* = 0.02), classical monocytes (*p* = 0.005), nonclassical (*p* = 0.003), and total granulocytes (*p* < 0.0001) frequencies were decreased, except for CD8+ T cells (*p* < 0.0001), which were increased in the PLHIV‐1 compared to the control participants. No significant changes were observed in the frequencies of total monocytes, eosinophils, and neutrophil cells between the two groups (Figure 1A).

FIGURE 1(A) Frequencies of cell subpopulations in the control and HIV‐1‐infected groups. (A) shows the frequencies of CD4 T cells (a), CD8 T cells (b), NK cells (c), NKT cells (d), B cells (e), total monocytes (f), classical monocytes (g), intermediate monocytes (h), nonclassical monocytes (i), total granulocytes (j), eosinophils (k), and neutrophils (l) in the control group (*n* = 20) and the people living with HIV‐1 (PLHIV‐1) (*n* = 90). Data shown as box plots representing medians (with 25th and 75th percentiles). The statistical differences were determined using the nonparametric Mann–Whitney test. Differences were considered significant when *p* values are less than 0.05. NK: natural killer, NKT: natural killer T, CD: cluster of differentiation. (B) Serum cytokine concentrations in the control and HIV‐1‐infected groups. (B) The concentration of IFN‐γ (a), TNF‐α (b), IL‐5 (c), IL‐6 (d), IL‐4 (e), IL‐7 (f), and IL‐13 (g) in the healthy control group (*n* = 20) and the people living with HIV‐1 (PLHIV‐1) (*n* = 90). Data shown as histograms representing medians with interquartile ranges. The statistical differences were determined using the nonparametric Mann–Whitney test. Differences were considered significant when *p* values are less than 0.05. IFN‐γ: interferon‐gamma, TNF: tumor necrosis factor, IL: interleukin.

**Figure:**
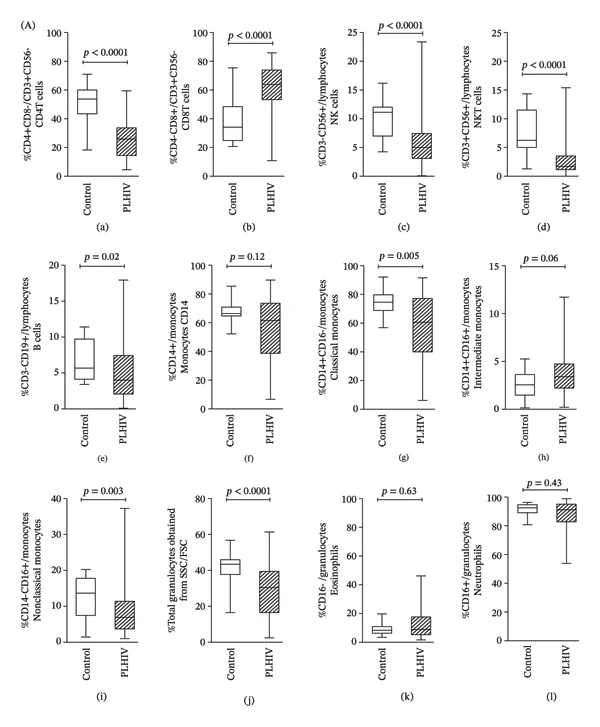

**Figure:**
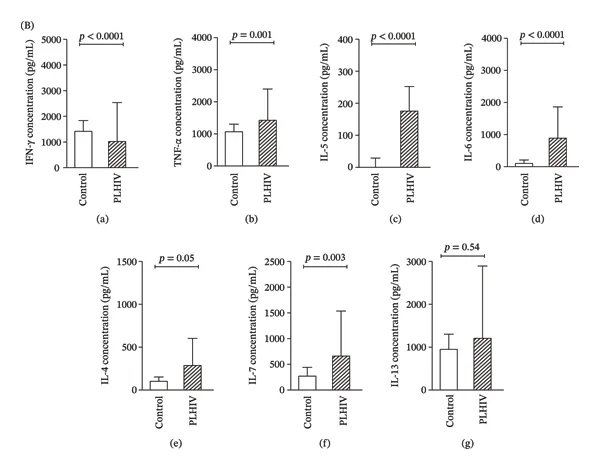

Regarding the cytokines, the global analysis results showed a lower concentration of IFN‐γ (*p* < 0.0001), and higher concentrations of TNF‐α (*p* = 0.001), IL‐5 (*p* < 0.0001), IL‐6 (*p* < 0.0001), and IL‐7 (*p* = 0.003) in PLHIV‐1, irrespective to the treatment line, than in the control participants. IL‐4 concentration was also increased in HIV‐1‐positive participants (*p* = 0.05) compared to the control participants. No significant difference in IL‐13 concentration was observed between the two groups (Figure 1B).

### 4.3. Immune Cell Frequencies and Cytokine Concentration According to the Treatment Lines in PLHIV‐1 Compared to the Control Participants

Considering treatment lines, we observed similar trends to those described above. Except for B cells, which did not change, there were decreases in CD4+ T cells, NK cells, NKT cells, classical monocytes, nonclassical monocytes, and total granulocyte frequencies, and increases in CD8+ T cells in PLHIV‐1 treated with both DTG and EFV regimens, compared to control individuals. No statistically changes were observed in total monocytes, eosinophils, and neutrophil cells. Overall, there were no significant changes in immune cell frequencies between HIV‐1‐positive individuals on both DTG and EFV treatment lines, Figure 2A.

FIGURE 2(A) Frequencies of cell subpopulations in the control and treatment line subgroups. (A) shows the frequencies of CD4 T cells (a), CD8 T cells (b), NK cells (c), NKT cells (d), B cells (e), total monocytes (f), classical monocytes (g), intermediate monocytes (h), nonclassical monocytes (i), total granulocytes (j), eosinophils (k), and neutrophils (l) in the control group (*n* = 20), DTG‐based treatment line (*n* = 38) and EFV‐based treatment line (*n* = 52). Data shown as box plots representing medians (with 25th and 75th percentiles). The statistical differences were determined using the nonparametric Kruskal–Wallis test with Dunn’s multiple comparisons. Differences were considered significant when *p* values were less than 0.05. NK: natural killer, NKT: natural killer T, CD: cluster of differentiation. (B) Serum cytokine concentrations in the control group and HIV‐1‐infected treatment line subgroups. (B) shows the concentration of IFN‐γ (a), TNF‐α (b), IL‐5 (c), IL‐6 (d), IL‐4 (e), IL‐7 (f), and IL‐13 (g) in the control group (*n* = 20), DTG‐based treatment line (*n* = 38), and EFV‐based treatment line (*n* = 52). Values are medians ± IQR. The statistical differences were determined using the nonparametric Kruskal–Wallis test with Dunn’s multiple comparisons. Differences were considered significant when *p* values were less than 0.05. IFN‐γ: interferon‐gamma, TNF: tumor necrosis factor, IL: interleukin.

**Figure:**
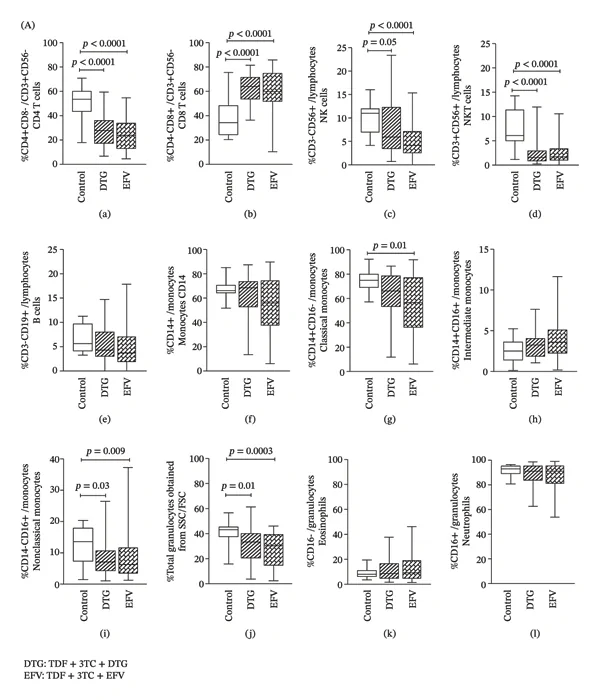

**Figure:**
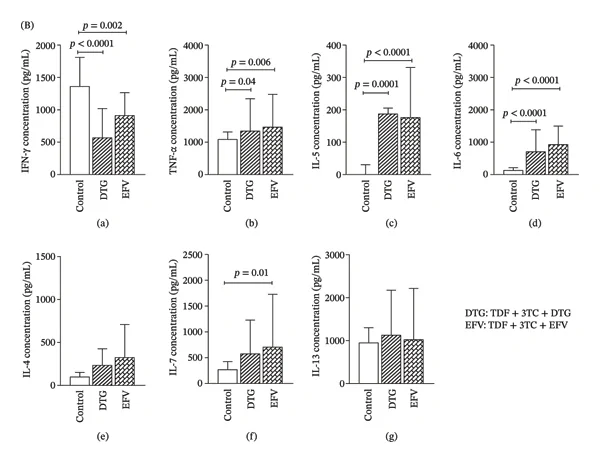

Similar trends to those observed when considering all PLHIV‐1 combined groups were found. HIV‐1‐positive participants under DTG or EFV regimen exhibited a low concentration of IFN‐γ and high concentrations of TNF‐α, IL‐5, IL‐6, and IL‐7 compared to the control participants (Figure 2B). No significant difference in IL‐13 concentration was observed between the HIV‐1‐positive participants under both regimens, compared to controls (Figure 2B). HIV‐1‐positive participants fed on both treatment lines did not show any significant changes in cytokine concentrations (Figure 2B).

### 4.4. Immune Cell Frequencies and Cytokine Concentrations According to Treatment Lines and Outcomes in HIV‐1‐Positive Groups and Control Participants

At the third level of comparison, taking into account the treatment line as well as treatment outcome, we observed that the frequencies of CD4+ T cells (*p* = 0.001, *p* = 0.01), and NKT cells (*p* < 0.0001, *p* = 0.003) were significantly decreased in PLHIV‐1, respectively, in failure and successful treatment groups under the DTG regimen, compared to the control. A reduced NK cell frequency was observed only in treatment‐failure group under DTG, compared to the controls. Considering the EFV treatment line, CD4+ T cells (*p* < 0.0001), NK cells (*p* = 0.0002), and NKT cells (*p* < 0.0001) frequencies were lower only in patients with treatment failure compared to control participants. In parallel, CD8+ T‐cell frequencies were significantly higher in PLHIV‐1 both in failure (*p* = 0.004) and successful (*p* = 0.004) treatment groups under the DTG regimen and were only elevated in the treatment‐failure group under EFV (*p* < 0.0001) compared to the control participants. Importantly, differences in CD4^+^ and CD8^+^ T‐cell frequencies were observed between treatment failure and treatment success under EFV, with higher CD4^+^ T‐cell frequencies (*p* = 0.0004) and lower CD8^+^ T‐cell frequencies (*p* = 0.004) in the treatment‐success group than in the failure groups. B‐cell frequencies did not differ significantly in any PLHIV‐1 group compared to control participants (Figure 3A).

FIGURE 3(A) Frequencies of lymphocyte subpopulations in the study groups. (A) shows the frequencies of CD4 T cells (a), CD8 T cells (b), NK cells (c), NKT cells (d), and B cells (e) in the control group (*n* = 20) and in people living with HIV‐1 (PLHIV‐1) who were on two ARV treatment regimens. Twenty‐five PLHIV‐1 on the dolutegravir regimen (TDF + 3TC + DTG) were at successful treatment. Thirteen PLHIV‐1 on DTG regimen were in failed treatment. Fifteen PLHIV‐1 on the efavirenz regimen (TDF + 3TC + EFV) were at successful treatment and thirty‐seven PLHIV‐1 on the EFV regimen were in failed treatment. Healthy control participants and PLHIV‐1 under ARV treatment for at least 6 months were recruited at the University Hospital of Abomey‐Calavi/Sô‐Ava (Benin) between October 2021 and April 2022. Data shown as box plots representing medians (with 25th and 75th percentiles). The statistical differences were determined using the nonparametric Kruskal–Wallis test with Dunn’s multiple comparisons. Differences were considered significant when *p* values were less than 0.05. NK: natural killer, NKT: natural killer T, CD: cluster of differentiation. (B) Frequencies of monocytes and polymorphonuclear subpopulations in the study groups. (B) shows the frequencies of monocytes CD14 (a), classical monocytes (b), intermediate monocytes (c), nonclassical monocytes (d), granulocytes (e), eosinophils (f), and neutrophils (g) in the control group (*n* = 20) and in people living with HIV‐1 (PLHIV‐1) who were on two ARV treatment regimens. Twenty‐five PLHIV‐1 on dolutegravir regimen (TDF + 3TC + DTG) were at successful treatment. Thirteen PLHIV‐1 on DTG regimen were at failure treatment. Fifteen PLHIV‐1 on efavirenz regimen (TDF + 3TC + EFV) were at successful treatment and thirty‐seven PLHIV‐1 on EFV regimen were at failed treatment. Healthy control participants and PLHIV‐1 under ARV treatment for at least 6 months were recruited at the University Hospital of Abomey‐Calavi/Sô‐Ava (Benin) between October 2021 and April 2022. Data shown as box plots representing medians (with 25th and 75th percentiles). The statistical differences were determined using the nonparametric Kruskal–Wallis test with Dunn’s multiple comparisons. Differences were considered significant when *p* values were less than 0.05. (C) Serum cytokine concentrations in the study groups. (C) The concentrations of cytokines, IFN‐γ (a), TNF‐α (b), IL‐5 (c), IL‐6 (d), IL‐4 (e), IL‐7 (f), and IL‐13 (g) in the control group (*n* = 20) and in people living with HIV‐1 (PLHIV‐1) who were on two ART regimens. Twenty‐five PLHIV‐1 on the dolutegravir regimen (TDF + 3TC + DTG) were at successful treatment. Thirteen PLHIV‐1 on DTG regimen were at failure treatment. Fifteen PLHIV‐1 on the efavirenz regimen (TDF + 3TC + EFV) were at successful treatment and thirty‐seven PLHIV‐1 on EFV regimen were at failed treatment. Healthy control participants and PLHIV‐1 under ARV treatment for at least 6 months were recruited at the University Hospital of Abomey‐Calavi/Sô‐Ava (Benin) between October 2021 and April 2022. Data are shown as histograms representing medians with interquartile ranges. The statistical differences were determined using the nonparametric Kruskal–Wallis test with Dunn’s multiple comparisons. Differences were considered significant when *p* values were less than 0.05. IFN‐γ: interferon‐gamma, TNF: tumor necrosis factor, IL: interleukin.

**Figure:**
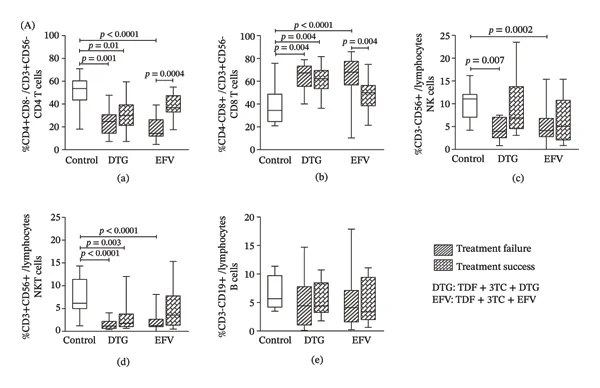

**Figure:**
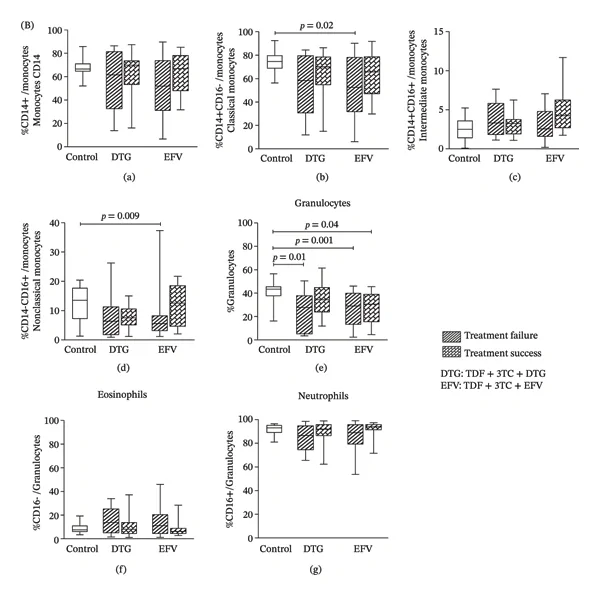

**Figure:**
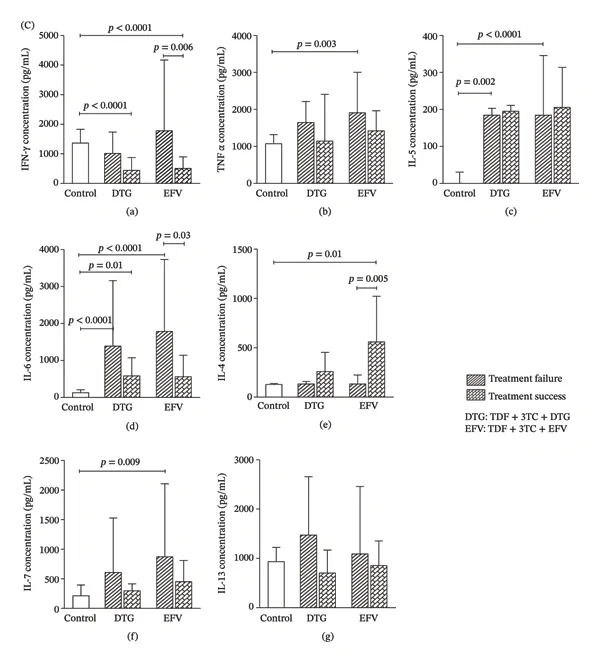

Regarding the frequencies of monocytes and granulocytes, the total monocyte frequency did not change, in PLHIV‐1 compared to control participants, regardless of treatment line or outcome. However, classical and nonclassical monocytes showed significantly lower frequencies in PLHIV‐1 who failed EFV treatment. The percentage of total granulocytes was found to be lower in the treatment‐failure groups under DTG (*p* = 0.01) and under EFV (*p* = 0.001), and in the treatment‐success group under EFV (*p* = 0.04). The percentages of eosinophils and neutrophils did not vary, regardless of treatment line or outcome, in PLHIV‐1 compared to control participants (Figure 3B).

We observed a low concentration of IFN‐γ in the treatment‐success group under DTG (*p* < 0.0001) and under EFV (*p* < 0.0001), compared to the control participants. The concentration of IFN‐γ was higher in the treatment‐failure group compared to the success group under DTG, but remains statistically significant in EFV treatment group (*p* = 0.006). The TNF‐α levels were significantly higher in treatment‐failure group under EFV (*p* = 0.003), compared to the control group. Compared to the control, the IL‐5 levels were significantly higher in treatment failure under DTG (*p* = 0.002) and under EFV (*p* < 0.0001). The IL‐6 cytokine levels were higher in treatment failure and success groups under the DTG regimen (*p* < 0.0001, *p* = 0.01, respectively), and in the treatment‐failure group under EFV (*p* < 0.0001). When comparing IL‐6 levels between success and failure in each treatment line group, the concentration was higher in the treatment‐failure group (*p* = 0.03) than in those under EFV.

The IL‐4 levels were significantly higher only in the EFV treatment‐success group than in the control group (*p* = 0.01). Additionally, the IL‐4 concentration was higher in the treatment‐success group than in the treatment‐failure group (*p* = 0.005) under the EFV regimen.

We observed that the IL‐7 level was significantly increased only in the EFV treatment‐failure group (*p* = 0.009) compared to the control participant group (Figure 3C). No significant difference was observed in IL‐13 levels (Figure 3C).

### 4.5. Factors Associated With Treatment‐Failure Risk in PLHIV‐1 on ART

The sociodemographic and immunological factors were analyzed for association with the risk of failure following treatment. As shown in Table 2, no risk of therapeutic failure was linked to sex, age, duration of treatment, occupation, or marital status. However, treatment with EFV regimen (TDF + 3TC + EFV) exhibited a significant risk of therapeutic failure (OR = 1.44, 95% CI, [1.18–1.75], *p* = 0.0003) compared to the treatment with DTG regimen (TDF + 3TC + DTG).

**TABLE 2:** Sociodemographic factors associated with antiretroviral therapeutic failure risk in PLHIV‐1.

Factors	Participants’ characteristics	Calculated risks associated with antiretroviral treatment failure			
		OR	*p* value	AOR	*p* value
		[_95%_CI (OR)]		[_95%_CI (OR)]	
Sex category	Female	1		1	
	Male	0.95 [0.75–1.20]	0.67	1.00 [0.79–1.25]	0.99

Age	≤ median (41 years)	1		—	
	> median	0.99 [0.98–1.00]	0.12	—	—

ARV treatment	TDF + 3TC + DTG	1		—	
	TDF + 3TC + EFV	1.44 [1.18–1.75]	0.0003	—	—

Duration of treatment	≤ median (42 months)	1		1	
	> median	1.12 [0.88–1.42]	0.33	0.96 [0.75–1.23]	0.33

Occupation	Artisan	1		1	
	Official	0.89 [0.69–1.15]	0.38	0.89 [0.70–1.13]	0.36
	Trader	0.81 [0.61–1.07]	0.15	0.84 [0.65–1.10]	0.22

Marital status	Single	1		1	
	Married	1.01 [0.72–1.42]	0.82	0.96 [0.68–1.34]	0.80
	Divorced	1.07 [0.74–1.55]	0.90	1.04 [0.73–1.49]	0.93
	Widower	0.98 [0.67–1.44]	0.67	1.02 [0.68–1.52]	0.92

*Note:* People living with HIV‐1 were on two ART regimens. Twenty‐five PLHIV‐1 on the dolutegravir regimen (TDF + 3TC + DTG) were at successful treatment with undetectable viral load (< 40 copies/mL); thirteen PLHIV‐1 on DTG regimen were at failure treatment (viral load > 1000 copies/mL); fifteen PLHIV‐1 on efavirenz regimen (TDF + 3TC + EFV) were at successful treatment; and thirty‐seven PLHIV‐1 on EFV regimen were at failure treatment. Ages and ARV treatment were included in the model for multivariate analysis. Patients were divided into two groups according to the parameter’s values below the medium (low) and values above the medium (high). *p* values less than 0.05 were considered as statistically significant. Analyses were performed only on PLHIV‐1 (*n* = 90).

Abbreviations: AOR = adjusted odds ratio, CI = confidence interval, OR = odds ratio.

In Table 3, we reported the results of therapeutic failure risk associated with cytokine levels in PLHIV‐1 on EFV and DTG regimens. We observed that an increased concentration of proinflammatory cytokines (IFN‐γ, TNF‐α, IL‐6, and IL‐7) was associated with high risk of treatment failure in PLHIV‐1 under the EFV regimen (OR = 1.61, 95% CI [1.22–2.13], *p* = 0.001 for IFN‐γ), (OR = 1.43, 95% CI [1.09–1.87], *p* = 0.01 for TNF‐α), (OR = 1.52, 95% CI [1.13–2.05], *p* = 0.008 for IL‐6), and (OR = 1.61, 95% CI [1.17–2.22], *p* = 0.005 for IL‐7). Only the proinflammatory IL‐7 cytokine was associated with a risk of treatment failure when treated with the DTG regimen (OR = 1.46, 95% CI [1.01–2.13], *p* = 0.05). When considering the anti‐inflammatory cytokines, a high IL‐4 concentration was associated with a low risk of treatment failure in PLHIV‐1 under the EFV regimen (OR = 0.41, 95% CI [0.30–0.58], *p* = 0.02 or DTG (OR = 0.64, 95% CI [0.51–0.93], *p* = 0.03). No association between IL‐5 or IL‐13 and the risk of treatment failure was observed.

**TABLE 3:** Therapeutic failure risk associated with cytokine levels in PLHIV‐1 on ART.

Cytokine concentrations (≤ and > median)		Calculated risks associated with antiretroviral treatment failure			
		TDF + 3TC + EFV	*p* value	TDF + 3TC + DTG	*p* value
		AOR [_95%_CI (OR)]	—	AOR [_95%_CI (OR)]	—
IFN‐γ	≤ median (738.20 ng/μL)	1	—	1	—
	> median	1.61 [1.22–2.13]	0.001	1.22 [0.88–1.68]	0.23

TNF‐α	≤ median (1432.69 ng/μL)	1	—	1	—
	> median	1.43 [1.09–1.87]	0.01	1.18 [0.86–1.60]	0.30

IL‐6	≤ median (847.59 ng/μL)	1	—	1	—
	> median	1.52 [1.13–2.05]	0.008	1.42 [0.99–2.04]	0.06

IL‐7	≤ median (409.14 ng/μL)	1	—	1	—
	> median	1.61 [1.17–2.22]	0.005	1.46 [1.01–2.13]	0.05

IL‐4	≤ median (146.67 ng/μL)	1	—	1	—
	> median	0.41 [0.30–0.58]	0.02	0.64 [0.44–0.93]	0.03

IL‐5	≤ median (177.21 ng/μL)	1	—	1	—
	> median	0.99 [0.84–1.17]	0.95	0.90 [0.51–1.59]	0.74

IL‐13	≤ median (901.75 ng/μL)	1	—	1	—
	> median	1.03 [0.80–1.32]	0.81	1.07 [0.77–1.49]	0.67

*Note:* People living with HIV‐1 were on two ART regimens. Twenty‐five PLHIV‐1 on the dolutegravir regimen (TDF + 3TC + DTG) were at successful treatment with undetectable viral load (< 40 copies/mL); thirteen PLHIV‐1 on DTG regimen were at failure treatment (viral load > 1000 copies/mL); fifteen PLHIV‐1 on efavirenz regimen (TDF + 3TC + EFV) were at successful treatment; and thirty‐seven PLHIV‐1 on EFV regimen were at failure treatment. Cytokine concentrations (ng/μL) expressed as numeric values allow for dividing patients into two groups according to the values below the medium (low) and values above the medium (high). *p* values less than 0.05 were considered as statistically significant. Age was included in the model for multivariate analysis. Analyses were performed only on PLHIV‐1 (*n* = 90).

Abbreviations: AOR = adjusted odds ratio, CI = confidence interval.

Regarding immune cells, we observed that a high frequency of CD4+ T cells was associated with a low risk of treatment failure in PLHIV‐1 on EFV regimen (AOR = 0.58, 95% [0.47–0.72], *p* < 0.0001), whereas high frequency of CD8+ T cells was associated with a high risk of treatment failure in PLHIV‐1 on EFV regimen (AOR = 1.57, 95% [1.25–1.98], *p* = 0.0003) (Table 4). While high proportions of eosinophils were significantly associated with a high risk of treatment failure in PLHIV‐1 on EFV regimen (AOR = 1.38, 95% [1.07–1.79], *p* = 0.01); this risk significantly decreased with the increase of neutrophils frequencies in PLHIV‐1 on EFV and DTG regimens respectively (AOR = 0.73, 95% [0.98–1.00], *p* = 0.02) and (AOR = 0.68, 95% [ 0.48–0.96], *p* = 0.04).

**TABLE 4:** Therapeutic failure risk associated with immune cell frequencies in PLHIV‐1.

Cell frequencies (≤ and > median)		Calculated risks associated with antiretroviral treatment failure			
		TDF + 3TC + EFV	*p* value	TDF + 3TC + DTG	*p* value
		AOR [_95%_CI (OR)]	—	AOR [_95%_CI (OR)]	—
CD4 T cells	≤ median (25.5%)	1	—	1	—
	> median	0.58 [0.47–0.72]	< 0.0001	0.79 [0.55–1.14]	0.22

CD8 T cells	≤ median (62.1%)	1	—	1	—
	> median	1.57 [1.25–1.98]	0.0003	1.06 [0.79–1.54]	0.72

NK cells	≤ median (5%)	1	—	1	—
	> median	0.98 [0.74–1.29]	0.90	0.73 [0.51–1.04]	0.10

NKT cells	≤ median (1.63%)	1	—	1	—
	> median	0.86 [0.66–1.11]	0.27	0.73 [0.52–1.03]	0.09

B cells	≤ median (4.02%)	1	—	1	—
	> median	1.16 [0.90–1.50]	0.24	0.95 [0.65–1.39]	0.80

CD14 monocytes	≤ median (61.45%)	1	—	1	—
	> median	0.84 [0.63–1.11]	0.23	0.82 [0.56–1.19]	0.30

Classical monocytes	≤ median (60.6%)	1	—	1	—
	> median	0.89 [0.66–1.18]	0.42	0.72 [0.50–1.03]	0.09

Intermediate monocytes	≤ median (3.43%)	1	—	1	—
	> median	0.82 [0.62–1.09]	0.19	1.08 [0.74–1.58]	0.67

Nonclassical monocytes	≤ median (6.85%)	1	—	1	—
	> median	0.84 [0.63–1.12]	0.24	0.93 [0.65–1.35]	0.73

Granulocytes	≤ median (30.7%)	1	—	1	—
	> median	1.05 [0.79–1.39]	0.70	1.03 [0.70–1.52]	0.86

Eosinophils	≤ median (8.82%)	1	—	1	—
	> median	1.38 [1.07–1.79]	0.01	1.33 [0.94–1.89]	0.11

Neutrophils	≤ median (91.2%)	1	—	1	—
	> median	0.73 [0.98–1.00]	0.02	0.68 [0.48–0.96]	0.04

*Note:* People living with HIV‐1 were on two ART regimens. Twenty‐five PLHIV‐1 on the dolutegravir regimen (TDF + 3TC + DTG) were at successful treatment with undetectable viral load (< 40 copies/mL); thirteen PLHIV‐1 on DTG regimen were at failure treatment (viral load > 1000 copies/mL); fifteen PLHIV‐1 on efavirenz regimen (TDF + 3TC + EFV) were at successful treatment; and thirty‐seven PLHIV‐1 on EFV regimen were at failure treatment. Cell frequencies expressed as percentages allow for dividing patients into two groups according to the values below the medium (low) and values above the medium (high). *p* values less than 0.05 were considered as statistically significant. Age is included in the model for multivariate analysis. Analyses were performed only on PLHIV‐1 (*n* = 90).

Abbreviations: AOR = adjusted odds ratio, CI = confidence interval.

## 5. Discussion

For several decades, one of the most effective approaches for biological monitoring of HIV infection has been regular surveillance of plasma VL and CD4^+^ T lymphocyte counts [27]. However, there is growing scientific consensus that therapeutic monitoring could be improved by identifying and validating additional easily accessible biological markers, particularly in low‐income countries [28]. In this context, the present study aimed to identify new immunological markers with prognostic value for HIV disease progression by evaluating the risks of therapeutic failure associated with immune cell frequencies and cytokine levels, in PLHIV‐1 receiving ARV regimens in Benin, TDF + 3TC + EFV or TDF + 3TC + DTG.

Regardless of the treatment line/outcome in HIV‐1‐infected participants, the overall analysis of the results showed a significant decrease in CD4+ T‐cell frequency, accompanied by a decrease in NK and NKT cell frequency in HIV‐1‐infected participants compared to control participants. Indeed, these cells play an important role in infection control through the production of IFN‐γ, which is necessary to induce infected cell lysis [29, 30]. The decrease in CD4+ T cells observed in HIV‐1‐infected individuals could be explained by the fact that these cells are the primary targets of viral replication [31]. HIV binds to the CD4 receptor, enters the cell, and uses the host’s replication machinery, ultimately leading to direct lysis of CD4+ T cells or apoptosis [32, 33]. Chronic immune activation further accelerates CD4+ T‐cell depletion through the death of uninfected cells. The reduction in NK and NKT cell frequencies in PLHIV‐1 may be related to persistent immune activation and viral‐mediated immune dysregulation. NK cells play a crucial role in early antiviral defense through cytotoxic activity and cytokine production, but chronic HIV‐1 infection has been shown to impair their maturation, reduce their cytolytic capacity, and alter their phenotypic distribution [34]. Similarly, NKT cells, which bridge innate and adaptive immunity, are susceptible to both functional exhaustion and numerical decline during chronic HIV infection, likely due to sustained antigenic stimulation, inflammatory cytokine milieu, and direct or indirect effects of viral proteins [35]. This depletion of NK and NKT cells may contribute to reduced innate immune responsiveness and facilitate ongoing viral persistence. Inversely, an increase of CD8+ T cells in both groups of PLHIV‐1, irrespective of treatment line or outcome, was observed. The general significant increase of CD8+ T cells, known as cytotoxic cells promoted by T helper‐1 cytokines, could be explained by the chronic immune activation and persistence of inflammation in PLHIV‐1 [36, 37]. No change was observed in eosinophil and neutrophil frequencies, regardless of treatment line or outcome, in PLHIV‐1 compared to controls, suggesting that HIV‐1 infection did not influence the frequencies of these cells. Overall, the observed decrease of B cells, classical and nonclassical monocytes and total granulocyte frequencies in PLHIV‐1 compared to control participants, irrespective of the outcome and line of treatment, confirmed the expected general immunodeficiency induced by HIV‐1 infection, cellular, and humoral immunity. In fact, the selective depletion of both classical and nonclassical subsets may reflect their recruitment to inflamed tissues, particularly lymphoid organs, and the endothelium, where they participate in antigen presentation and inflammatory responses [38].

In this study, the suspected chronic inflammation in PLHIV‐1, consistent with an increased frequency of CD8+ T cells, was confirmed by the behavior of cytokines in HIV‐1‐infected participants. Indeed, we observed elevated levels of proinflammatory cytokines, such as TNF‐α, IL‐5, IL‐6, and IL‐7 in HIV‐1‐positive participants, regardless of treatment type, compared to control participants. In fact, it has been shown that persistent HIV‐1 viral replication in tissues such as lymph nodes could lead to an increase in proinflammatory cytokines IL‐7 and TNF‐α [8]. Moreover, HIV infection induces an increase in IL‐5 levels, as it has also been reported by Shete et al. [39]. IL‐4, an anti‐inflammatory cytokine, increased, but not significantly, to reverse the effect of proinflammatory cytokines in these patients. The unchanged behavior of IL‐13, another anti‐inflammatory cytokine, contributed to maintaining the proinflammatory status of HIV‐infected patients. Indeed, HIV infection, by triggering high plasma VL, maintains a proinflammatory state in HIV‐1‐positive persons [40, 41].

Extensive analysis of the results, taking into account the treatment line and outcome on the frequencies of immune cells, showed that cellular immunity destroyed by HIV infection was significantly improved mainly in the successful EFV group. Humoral immunity was not affected in HIV‐1 patients, regardless of the treatment line or its outcome. These observations are linked to the fact that the frequencies of CD4+ T cells, NK cells, and NKT cells decreased significantly regardless of treatment outcome under DTG, while they were all restored in the successful EFV group. Only NK cells were restored in DTG‐success group. In fact, NK cell frequency would be positively correlated with therapeutic success under DTG and EFV regimens in HIV infection [42]. However, both regimens did not contribute to restore NKT cells [43]. At the same time, the proportions of CD8+ T cells, which were significantly disrupted in the DTG group, regardless of the outcome, and in EFV‐failure patients, were restored in the successful EFV group. The present results revealed no significant changes in B‐cell frequency in PLHIV under both regimens, as compared to control participants, suggesting that HIV infection did not influence the frequencies of these cells. Overall, the frequencies of all these cells were mainly restored in HIV patients successfully treated with EFV compared to patients under the DTG regimen or control participants.

As far as monocytes and granulocytes are concerned, no change was observed in the frequencies of total monocytes regardless of treatment line or outcome, in PLHIV‐1 compared to controls. However, only classical and nonclassical monocytes showed a slight but significant decrease in EFV‐failure group. The depletion of classical and nonclassical monocytes in HIV‐1‐infected individuals receiving the EFV treatment line may result from a combination of HIV‐1‐related immune dysregulation and treatment‐associated effects [38]. EFV has been reported to induce oxidative stress and mitochondrial dysfunction in mononuclear cells, potentially impairing monocyte survival [38].

In the present study, the percentage of eosinophils and neutrophils did not vary, regardless of treatment line or outcome, in HIV‐1‐positive participants compared to controls. In fact, neutrophils being involved in the primary immune response against opportunistic infections that appear during the HIV infection could help to maintain a relative innate immune response in these individuals [44]. However, the persistent viral replication characteristic of high VL associated with subsequent treatment failures could affect the frequencies, phenotypes, and functions of these cells during HIV‐1 infection [45].

By carefully analyzing the behavior of cytokines with respect to treatment lines and outcomes, we observed some interesting results. In our study, a decrease in the production of the key Th1 cytokine IFN‐γ was observed in participants who achieved successful viral suppression on EFV‐ and DTG‐based regimens, while no such decrease was detected in those with viral failure. This pattern suggests that effective ART contributes to reducing IFN‐γ levels primarily by suppressing HIV replication and the subsequent attenuation of chronic immune activation [46]. IFN‐γ is a key Th1 cytokine involved in antiviral defense, and its elevated levels in untreated or failing patients likely reflect ongoing antigenic stimulation due to persistent viral replication [46]. The concomitant increase in TNF‐α, IL‐6, IL‐5, and IL‐7, particularly in EFV participants who failed treatment, was consistent with chronic immune activation and dysregulation. TNF‐α and IL‐6 are proinflammatory cytokines produced by activated monocytes/macrophages and other immune cells in response to microbial translocation and residual viral replication [47]. Elevated IL‐4 levels indicate a bias toward a Th2 immune profile, often observed in advanced HIV infections and potentially further inhibiting Th1 responses. IL‐7 elevation is generally a compensatory homeostatic response to T‐cell depletion, as this cytokine promotes T‐cell survival and proliferation [47].

The profile of these immune parameters (cells and cytokines), depending on the therapeutic line and treatment outcome in volunteers, revealed that there would necessarily be an association between the evolution of these parameters and the therapeutic outcome of patients receiving ART. In other words, it seems obvious to predict the therapeutic efficacy of the ART through the profile of these immune parameters. This observation prompted us to calculate the risks of therapeutic failure or success associated with each treatment regimen, particularly EFV and DTG, as in the present study. This approach aimed to standardize a model for calculating the risks of therapeutic failure based on the evolution of immune parameters. This would represent a new approach to facilitate patient monitoring. To our knowledge, very few studies have addressed this aspect. In order to examine the risk of drug treatment failure associated with immune parameters, we performed a logistic regression test. Indeed, we observed that EFV‐based ART was associated with a higher risk of treatment failure than DTG‐based regimens. Several factors may explain this difference. DTG, as an integrase strand transfer inhibitor, has a higher genetic barrier to resistance than EFV, a NNRTI, making virological failure less likely even in the context of suboptimal adherence [48, 49]. Moreover, DTG achieves more rapid viral suppression and maintains potent antiviral activity against a broader range of HIV‐1 variants, which may reduce the likelihood of persistent low‐level replication and the emergence of resistance, compared to EFV [50].

We observed that high proinflammatory cytokines (IFN‐γ, TNF‐α, IL‐6, and IL‐7) were associated with a high risk of treatment failure in PLHIV‐1 on the EFV regimen, whereas only high levels of IL‐7 were associated with an increased risk of treatment failure in PLHIV‐1 on the DTG. However, high levels of anti‐inflammatory IL‐4 were associated with a low risk of therapeutic failure in PLHIV‐1 on EFV and DTG. Our results showed that higher IL‐4 levels appeared to be protective, as they were associated with a reduced risk of treatment failure, suggesting that higher levels of IL‐4 may reflect improved immune balance and successful treatment [8]. IL‐4, a Th2 cytokine, can downregulate proinflammatory cytokine production and limit excessive immune activation, potentially preserving immune function and slowing disease progression. These findings suggest that in EFV‐treated individuals, a heightened proinflammatory cytokine milieu is detrimental for treatment outcomes, whereas a shift toward a more regulated, anti‐inflammatory profile may be beneficial. Although treatment duration varied between patient groups, no significant differences were observed in cellular or cytokine proportions among HIV‐1‐infected participants, except for higher CD4+ T‐cell percentages and lower CD8+ T‐cell percentages in the EFV treatment‐success group compared with the treatment‐failure group, a pattern consistent with expected immune recovery. Therefore, treatment duration was unlikely to have materially influenced the overall comparisons.

In the EFV‐based treatment group, a higher percentage of CD4^+^ T cells was associated with a reduced risk of treatment failure, consistent with the central role of CD4^+^ T lymphocyte preservation in effective immune control of HIV replication. Adequate CD4^+^ T‐cell numbers support coordinated antiviral responses and enhance CD8^+^ T‐cell function [51], thereby improving treatment outcomes. Similarly, higher neutrophil percentages appeared protective, likely reflecting preserved innate immune competence and the ability to control opportunistic infections that can exacerbate immune activation and viral replication. In contrast, elevated CD8^+^ T cell and eosinophil percentages were, respectively, linked to an increased risk of treatment failure, which may reflect chronic immune activation and expansion of dysfunctional or exhausted cytotoxic T cells in the setting of persistent antigen exposure, or allergic‐type inflammation, or co‐infections (e.g., helminthiasis) that can divert immune resources away from effective antiviral activity. Collectively, these findings suggested that, under EFV‐based regimens, maintenance of robust CD4^+^ T cell and neutrophil levels appeared beneficial, whereas elevations in CD8^+^ T cells and eosinophils might serve as markers of pathological immune activation and poorer treatment outcomes.

We therefore concluded that only high levels of proinflammatory cytokines were associated with an increased risk of treatment failure in PLHIV‐1 on EFV. The risk of treatment failure was limited only to high IL‐7 levels in PLHIV‐1 on DTG. In fact, increased production of these proinflammatory cytokines was linked to continued immune activation and inflammation in PLHIV‐1, which would likely be the consequence of several mechanisms such as persistent HIV‐1 replication in the lymph nodes and gastrointestinal tract [8]. Furthermore, it has been reported that patients who developed DTG or EFV resistance mutations have had treatment failure [52, 53]. Such observations suggest that these cytokines were induced in response to high viremia or to mutations that would result from therapeutic failure. Our results corroborate those of other authors who have shown that IFN‐γ, TNF‐α, IL‐6, and IL‐7 were associated with a high burden of plasma VL [8, 54].

## 6. Conclusion

Despite progress made in recent years in the care and monitoring of people living with HIV‐1 (PLHIV‐1), the incidence of this disease remains high worldwide, highlighting the need of inventing new treatment and monitoring strategies. The present study identified new, readily accessible biological markers useful in the clinical monitoring of HIV infection, principally in resource‐limited countries. We observed a positive correlation between the risk of treatment failure and increased production of proinflammatory cytokines IFN‐γ, TNF‐α, IL‐6, and IL‐7, as well as a high frequency of CD8+ T cells in HIV‐1‐infected patients receiving ART based on EFV and DTG. Conversely, the risk of treatment failure appears to decrease with an increased number of CD4+ T cells and the anti‐inflammatory cytokine IL‐4. Thus, these cytokines and immune cells could serve as prognostic factors for monitoring the progression of HIV‐1 infection and the responses to ARV treatment in infected individuals, although further studies with larger sample sizes are needed to confirm our data.

## 7. Limitations

Our study did not lack limitations. First, the declarations of the state of health of the control patients did not benefit from verification elements. Second, the sample size was small and did not allow definitive conclusions. Small samples are more likely to contain random variations that do not accurately reflect the target population. A larger sample size reduces this variability and minimizes sampling error, making the results more reliable. Additionally, the intracellular cytokine staining, which would have been more informative and helpful to identify the cellular sources of cytokines, was not performed in the current study because of the limited resources for specific laboratory reagents.

## Author Contributions

Yaou Pierrot Assogba was in charge of the major technical aspects of the work and wrote the manuscript. Adefounke Prudencia Adechina and Odilon Paterne Nouatin participated in the technical work, contributed to analyzing data regarding the flow cytometry, and participated in the manuscript writing. Edmond Tchiakpe contributed to analyzing data regarding the VL and participated in the manuscript writing. Rufine Abossèdé Fachinan and Roukoyath Moyoriola Adegnika participated in the technical work. Euloge Sènan Adjou participated in the manuscript writing. Halimatou Diop‐Ndiaye contributed to the development of the protocol and in the manuscript writing. Honoré Sourou Bankole and Akadiri Yessoufou designed the study, supervised the work, participated in the manuscript writing, and established the collaborative aspects.

## Funding

No funding was received for this manuscript.

## Disclosure

All authors read and approved the final manuscript.

## Ethics Statement

This study was approved by the National Ethics Committee for Health Research of Benin (CNERS) under the No. 137 MS/DC/SGM/CNERS/SA dated November 24, 2021. The study was conducted in accordance with the Declaration of Helsinki 1964 (as revised in Edinburgh 2000). Informed signed consent from each eligible patient was obtained before inclusion. All information collected in this study has been treated and managed in strict confidence. Unique identifiers for each enrolled patient were used, and access to data was protected by an encrypted password. Only the attending physician can link this identifier to the patient for biological and medical follow‐up.

## Conflicts of Interest

The authors declare no conflicts of interest.

## Data Availability

The datasets used and/or analyzed during the current study are available from the corresponding author on reasonable request.
